# Effects of Dietary Supplementation with dl-Methionine and dl-Methionyl-dl-Methionine in Breeding Pigeons on the Carcass Characteristics, Meat Quality and Antioxidant Activity of Squabs

**DOI:** 10.3390/antiox8100435

**Published:** 2019-10-01

**Authors:** Shi-Guang Jiang, Neng-Xia Pan, Meng-Jie Chen, Xiu-Qi Wang, Hui-Chao Yan, Chun-Qi Gao

**Affiliations:** Guangdong Provincial Key Laboratory of Animal Nutrition Control/Key Laboratory of Chicken Genetics, Breeding and Reproduction, Ministry of Agriculture, College of Animal Science, South China Agricultural University, Guangzhou 510642, Guangdong, China; sgjiang@stu.scau.edu.cn (S.-G.J.); wyxie@stu.scau.edu.cn (N.-X.P.); mjchen@stu.scau.edu.cn (M.-J.C.); yanhc@scau.edu.cn (H.-C.Y.)

**Keywords:** squabs, dl-methionine, dl-methionyl-dl-methionine, carcass characteristics, meat quality, antioxidant activity

## Abstract

This study aimed to investigate the effects of dietary supplementation with dl-methionine (dl-Met) and dl-methionyl-dl-methionine (dl-Met-Met) in breeding pigeons on the carcass characteristics, meat quality and antioxidant activity of squabs. A total of 324 pairs of breeding pigeons were selected and allotted to 9 treatments in a completely randomized design, and the birds were fed dietary treatments for 45 d, including a Met-deficient basal diet (BD, crude protein = 15%, Met = 0.25%) and BD + 0.15%, 0.30%, 0.45%, or 0.60% dl-Met or dl-Met-Met diets. Compared with the diet fed to the BD group, dietary dl-Met or dl-Met-Met supplementation effectively increased the carcass yield, semieviscerated yield, eviscerated yield, breast muscle yield, thigh muscle yield, a* value, catalase activity, total superoxide dismutase activity and glutathione peroxidase activity, but decreased the L* value, malonaldehyde concentration, drip loss and cooking loss of squabs (*p* < 0.05). The relative bioavailability values of dl-Met-Met relative to those of dl-Met were 467% and 376% based on carcass yield and breast muscle yield, respectively (*p* < 0.001). Moreover, dl-Met-Met was more effective than dl-Met in decreasing the drip loss and improving the antioxidant activity of the breast and thigh muscles of squabs (*p* < 0.05). As a source of Met, dl-Met-Met, rather than dl-Met, was more beneficial to squabs.

## 1. Introduction

As a low-fat, high-protein meat source, pigeon meat accounts for a large portion of the meat consumed in Asia. In the past 5 years, there have been approximately 42 million pairs of breeding pigeons in China, and 680 million squabs sold every year; the total squab production in China accounts for more than 80% of global production. While pursuing greater quantities of meat products, consumers are also increasingly demanding higher meat quality. However, pigeons are altricial birds, and squabs do not have independent feeding ability after hatching. From shelling to marketing, the nutrients provided to squabs during growth come mainly from breeding pigeons (males and females). Thus, the compositions and nutrient levels of the diets of breeding pigeons play an important role in increasing pigeon crop milk production and young squab slaughter performance [1]. In addition, the consumption of meat products with increased malonaldehyde (MDA) levels affects human health and causes adverse changes to glycolysis. Undesirable flavors and odors are also formed [2,3]. Therefore, an effective method is needed to protect intramuscular lipids against oxidative damage and to enhance meat quality. Currently, nutrition regulation has become an important means to improve meat yield and muscle quality in poultry [4,5,6]. For example, adding appropriate amino acids to the diet can improve the growth and slaughter performance of pigeons [1].

dl-Methionine (dl-Met) is an indispensable amino acid in poultry nutrition because most poultry diets depend on corn and soybean meal as the main component, which necessitates dietary supplementation with dl-Met to satisfy nutritional requirements [7]. dl-Met is also a precursor of important intermediates, such as metabolic pathway components and glutathione, which have antioxidant characteristics, and which also decrease oxidative damage caused by lipid peroxidation [8,9]. This function is similar to that of selenium as a precursor of selenoprotein synthesis involved in the antioxidant defense mechanism of the body [10,11]. A previous study has suggested that deficiency in dl-Met consumption has a negative impact on animals, such as growth inhibition and the induction of metabolic disorder [7]. Supplementation of the poultry diet with dl-Met has been associated with the tendency to have more muscle production [12] and to improve growth performance and meat quality [13]. Moreover, adding appropriate dietary dl-Met has been indicated to improve the color of muscle and decrease the shear force [14]. A previous study also demonstrated that dietary supplemental dl-Met levels should be higher than the value recommended by the National Research Council (NRC) (1994) to improve the redox status of turkeys [15]. More Met residues can be combined with reactive oxygen species in the body to form two Met sulfoxide isomers, which regenerate Met under the action of Met sulfoxide reductase. This process can eliminate oxidative protein damage caused by reactive oxygen species [16].

In addition, small peptides have received increasing attention as a new source of nutrients, such as dl-methionyl-dl-methionine (dl-Met-Met). Relevant studies have shown that dl-Met-Met is mainly transported and absorbed by the oligopeptide transporter 1 (PepT1) and oligopeptide transporter 2 (PepT2) transporters in the intestinal epithelium, which does not occupy the absorption channels of other monomeric amino acids and is not easily saturated [17,18]. dl-Met-Met was also found to be more bioavailable and more efficient than dl-Met [19,20]. Although some functions of dl-Met-Met are similar to those of monomeric dl-Met and the overall effect is better, whether dl-Met-Met also acts as a precursor of antioxidant substances to improve muscle quality warrants further study. We hypothesized that the addition of dl-Met-Met or dl-Met could improve the antioxidant activity in the muscle, thereby promoting the meat production and meat quality of squabs, and that the effect of dl-Met-Met was better than that of dl-Met. Therefore, the objective of this study was to evaluate the carcass characteristics, meat quality and antioxidant activity of squabs with the addition of dl-Met or dl-Met-Met in the breeding pigeon diet.

## 2. Materials and Methods

All methods and management procedures used in this study were complied with the guidelines established by South China Agricultural University (Guangzhou, China), and experiments were approved by the Animal Ethics Committee of South China Agricultural University (Guangzhou, China).

### 2.1. Materials

dl-Met (>99% purity) and dl-Met-Met (>95% purity) were obtained from Evonik Degussa (China) Co., Ltd. (Beijing, China); sodium chloride was purchased from Sigma-Aldrich (Milan, Italy); all commercial colorimetric kits were purchased from Jiancheng (Nanjing, China). The instruments included a handheld colorimeter (CR-410, MINOLTA, Japan), tenderness tester (CLM3B, Tenovo, Beijing, China), automatic sample rapid grinding machine (JXFSTPRP-32, Jingxin, Shanghai, China), electronic balance (LS120A, Precisa, Switzerland), high-speed refrigerated centrifuge (5810R, Eppendorf, Germany), and spectrometer (721, Guangxue, Shanghai, China).

### 2.2. Animals, Experimental Design and Diets

A total of 324 pairs (324 males and 324 females) of 3-year-old white king breeding pigeons with similar reproductive performance and body weight were obtained from the WENS Foodstuff Group Co., Ltd (Yingde, China). Pigeons were randomly divided into nine treatments, with six replicates (each replicate containing 6 pairs) per treatment. Each pair of breeding pigeons with 4 young squabs were housed in a man-made cage with a nest and a perch. Nine experimental diets (Table 1) were formulated (basal diet [BD], 0.15% dl-Met, 0.30% dl-Met, 0.45% dl-Met, 0.60% dl-Met, 0.15% dl-Met-Met, 0.30% dl-Met-Met, 0.45% dl-Met-Met, 0.60% dl-Met-Met). The BD was formulated without any additional methionine addition, and the other eight diets were supplemented with four graded levels of methionine (0.15%, 0.30%, 0.45%, and 0.60%) with two different methionine sources (dl-Met and dl-Met-Met).

### 2.3. Management

Each pair of breeding pigeons was raised in an open dovecote, which was ventilated both mechanically and naturally, with a photoperiod of 16 h of daily light. During the experiment, the trough, water cup and floor were cleaned and disinfected regularly. Breeding pigeons were allowed free access to food and water with adequate health care sand. The experimental period was 45 d (including an incubation period of 17 d and a lactation period of 28 d). At the end of the experiment, 36 squabs from each treatment were selected randomly for the carcass characteristics, and meat quality was evaluated.

### 2.4. Sample Collection

At the end of the experiment, 36 squabs from each treatment were randomly selected and weighed after feed deprivation for 12 h. Squabs were bled from the jugular vein, euthanized by cervical dislocation and necropsied immediately. Then, samples were immediately collected from the right breast muscles and thigh muscles and stored at 4 °C for subsequent determination of meat quality (including meat color, drip loss, cooking loss and shear force). The breast and thigh muscles were quickly frozen in liquid nitrogen and stored at −80 °C for antioxidant activity and further analyses.

### 2.5. Carcass Characteristics

At the end of the experiment, squabs were fasted for 12 h and then humanely slaughtered. After blood and feathers were removed, carcasses were weighed to calculate carcass yield. The breast muscles (pectoralis major) and thigh muscles (all the muscles on the surface of the thigh bone) were removed and weighed without bones. At the same time, the other organs of squabs were extracted and weighed. Carcass yield was calculated as percentage of live weight, eviscerated yield and semieviscerated yield were calculated as percentages of slaughtering weight, and breast muscle yield and thigh muscle yield were calculated as percentages of eviscerated weight [21,22].

### 2.6. Meat Quality Assay

At 24 h after slaughter, the color of the breast muscle was evaluated (measured three times at three different locations around the meat sample and averaged) using a handheld colorimeter based on the CIELAB system (L* = lightness; a* = redness; b* = yellowness). Next, the drip loss and cooking loss of samples were assayed at 24 h postmortem, as in a previous trial [23,24]. Briefly, meat samples were weighed, hung in a sealed plastic bag, and stored at 4 °C for 24 h. Then, samples were weighed again to calculate drip loss. The samples were weighed and packed in a sealed plastic bag to be cooked by immersion in a water bath to an internal temperature of 75 °C, which was maintained for 30 min. Subsequently, the samples were weighed again to calculate cooking loss. For the shear force determination, which was measured with the tenderness tester after the muscle samples were cooked, the average of the three measurements of each sample was used as the shear force value [25].

### 2.7. Assay of Antioxidant Activities in Muscle

Muscle samples of approximately 200 mg were homogenized with ice-cold sodium chloride solution by an Automatic Sample Rapid Grinding Machine. Then, the homogenization buffer was centrifuged at 3,500 rpm/min for 10 min at 4 °C. The supernatant was used for assaying the antioxidant indices, including the activities of catalase (CAT), total superoxide dismutase (T-SOD), and glutathione peroxidase (GSH-Px), the total antioxidant capacity (T-AOC), and the concentration of MDA. These parameters were determined by using a commercial colorimetric kit. Briefly, the absorbance of each sample was spectrophotometrically measured using a spectrometer against a blank. Finally, the activity was calculated according to the instructions provided by the manufacturer [25].

### 2.8. Statistical Analysis

All data were analyzed with one-way ANOVA using the PROC-GLM procedure of SAS software (SAS Institute Inc., Cary, NC). Primary and quadratic linear trend analyses of various indices at different concentrations and Met source levels were performed by the orthogonal polynomial comparison method. The sources of Met were the main effects of the statistical model. The results are presented as the means ± SEM, and the differences among treatments were considered statistically significant if *p* < 0.05. Furthermore, to calculate the relative bioavailability of dl-Met-Met to dl-Met, a multiexponential regression equation was used to assess the efficacy of dl-Met-Met relative to that of dl-Met [26]. The following model was used to assess bioavailability: y = A + B × {1 – exp[−(C_1_X_1_ + C_2_X_2_)]}, where y is the response variable, A is the common intercept, A + B is common asymptote, C_1_ is the curvature steepness coefficient for dl-Met, C_2_ is the curvature steepness coefficient for dl-Met-Met, and X_1_ and X_2_ are the contents of dl-Met and dl-Met-Met present in the diet, respectively.

## 3. Results

### 3.1. Carcass Characteristics

The carcass characteristics of squabs are shown in Table 2. Compared with the diet fed to the BD group, breeding pigeon dietary supplementation with 0.15%, 0.30%, 0.45% and 0.60% dl-Met or dl-Met-Met significantly increased the carcass yield, semieviscerated yield, eviscerated yield, breast muscle yield and thigh muscle yield of squabs (*p* < 0.05). Furthermore, the results of the trend analysis indicated that the supplemental dl-Met or dl-Met-Met in the breeding pigeon diet showed a linear or quadratic curve trend to increase the carcass yield and breast muscle yield of squabs (*p* < 0.05).

### 3.2. Relative Bioavailability

**Figure 1:**
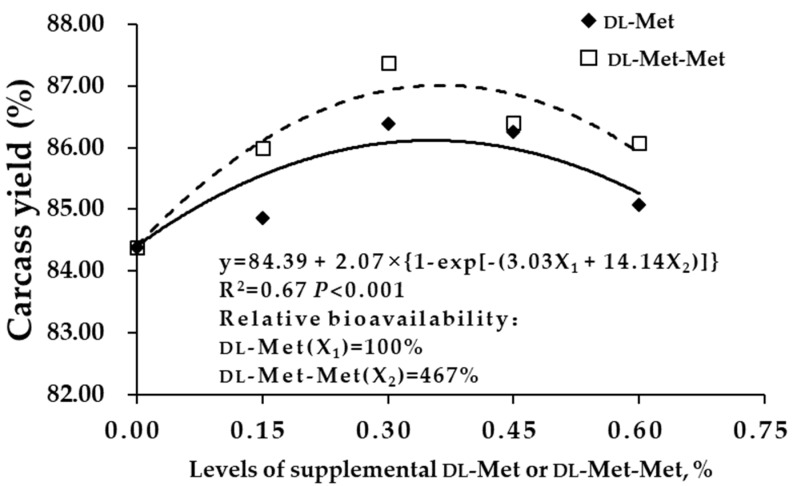
Relative bioavailability of dl-Met-Met to dl-Met based on the carcass yield of young squabs (n = 36). y = 84.39 + 2.07 × {1 – exp[−(3.03X_1_ + 14.14X_2_)]}, (y: carcass yield; X_1_: dl-Met; X_2_: dl-Met-Met). The relative bioavailability of dl-Met-Met was 467% at an equal concentration (dl-Met: dl-methionine; dl-Met-Met: dl-methionyl- dl-methionine). )

**Figure 2:**
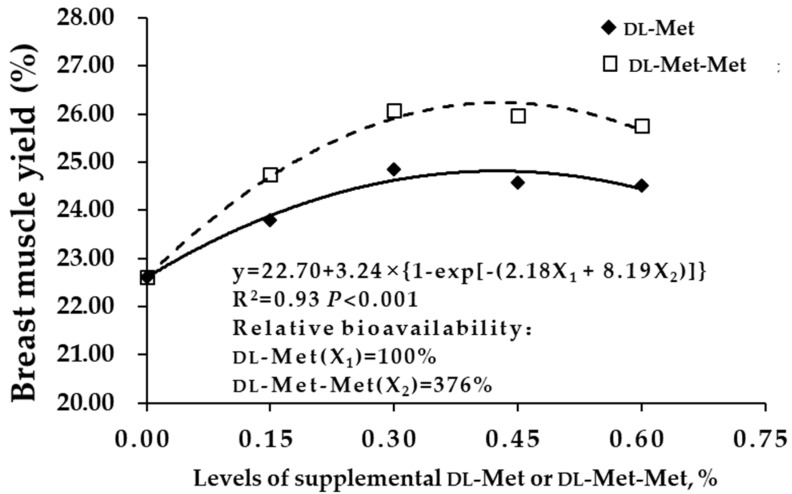
Relative bioavailability of dl-Met-Met to dl-Met based on the breast muscle yield of young squabs (n = 36). y = 22.70 + 3.24 × {1 – exp[−(2.18X_1_ + 8.19X_2_)]}, (y: breast muscle yield; X_1_: dl-Met; X_2_: dl-Met-Met). The relative bioavailability of dl-Met-Met was 376% at an equal concentration (dl-Met: dl-methionine; dl-Met-Met: dl-methionyl-dl-methionine).

The carcass yield and breast muscle yield of squabs were fitted to a multiexponential regression equation (Figure 1 and Figure 2). The relative biological values of dl-Met-Met relative to those of dl-Met were 467% and 376%, respectively, based on the carcass yield and breast muscle yield of squabs (*p* < 0.001). Furthermore, carcass yield gradually increased with the increase in the amount of dl-Met or dl-Met-Met added from different sources, and peaked when the added amount was 0.30%. 

### 3.3. Meat Quality

The results of meat quality traits, including muscle color, shear force, drip loss and cooking loss, are presented in Table 3. In comparison to the diet fed to the BD group, dl-Met or dl-Met-Met supplementation increased the a* value but decreased the L* value, drip loss and cooking loss in the breast muscle of squabs (*p* < 0.05). The drip loss of breast muscle at 24 h was significantly decreased under breeding pigeon dietary supplementation with dl-Met-Met, as compared with that under dietary supplementation dl-Met (*p* < 0.05). Furthermore, the results of trend analysis indicated that the supplemental dl-Met or dl-Met-Met in the breeding pigeon diet showed a linear or quadratic curve to increase the a* value and decrease the L* value of the breast muscle of squabs (*p* < 0.05).

### 3.4. Antioxidant Activity

In this experiment, the optimum supplementation with dl-Met or dl-Met-Met was 0.30% in the breeding pigeon diet, according to the carcass characteristics and meat quality improvement effect of squabs. The changes in the antioxidant activity of breast and thigh muscles were also measured. The results of the antioxidant activity of muscle are shown in Table 4 and Table 5. In comparison to the diet fed to the BD group, dietary dl-Met or dl-Met-Met supplementation effectively improved the CAT, T-SOD and GSH-Px activities in the muscles of squabs (*p* < 0.05). The activities of CAT, T-SOD and GSH-Px were higher in the muscles of the dl-Met-Met groups than in those of the dl-Met groups (*p* < 0.05). At the same time, the concentrations of MDA were significantly decreased in the groups supplemented with dl-Met and dl-Met-Met (*p* < 0.05).

## 4. Discussion

With the improvement of living standards, the single-minded pursuit of large quantities of meat products has been replaced by an interest in both the quantity and quality of meat [27]. This study was designed to explore the impacts of dl-Met-Met and conventional dl-Met on the carcass characteristics, meat quality and antioxidant activity of squabs, and to compare their bioavailability.

Carcass characteristics are indicators of nutrient deposition, and the meat yield of breast and thigh muscles is an important indicator of carcass characteristics in poultry. Previous studies have shown that dietary dl-Met levels should be higher than those recommended by the NRC (1994), which can increase the carcass yield and breast muscle yield of broilers [28,29,30,31,32]. However, the dietary supplementation of Met-Met on the carcass characteristics and meat quality has not been characterized. The present study indicated that breeding pigeon dietary supplementation with 0.15%–0.60% dl-Met or dl-Met-Met significantly increased the carcass yield, semieviscerated yield, eviscerated yield, breast muscle yield and thigh muscle yield of squabs, as compared with the diet fed to the BD group. The relative biological values of dl-Met-Met relative to those dl-Met were 467% and 376% based on the carcass yield and breast muscle yield of squabs, respectively. This result was consistent with the findings by Niu et al. [26], who found that Met-Met was more utilizable than dl-Met, with calculated relative bioavailability values of 286%, 276% and 300% based on weight gain, growth rate and feed efficiency, respectively. This finding may be attributable to the transport and absorption of dl-Met-Met, which does not occupy the absorption channels of other monomeric amino acids and is not easily saturated [17]. This finding may also be attributable to the differences in solubility and rates of the Met sources across the brush border membrane [33]. Overall, appropriate supplementation with Met or Met-Met in the diet can improve the carcass characteristics of poultry [34].

The quality of pigeon meat includes sensory quality and edible quality, which are mainly affected by muscle color, drip loss, shear force and cooking loss. These characteristics are important quality parameters that affect a consumer’s purchasing decision [3,35]. In the present study, we found that the addition of dl-Met or dl-Met-Met increased a* values and inversely decreased L* values, drip loss and cooking loss. There was a significant difference in drip loss between the dl-Met-Met group and the other two groups. However, this supplementation had no significant effect on the shear force. dl-Met supplementation significantly increased a* values and decrease L* values, drip loss and cooking loss, which was in accordance with the results observed in a previous study [36].

The increase in a* values and decrease in L* values of muscle reflect an improvement of meat color, which indicates the enhancement of anti-lipid peroxidation activity [37]. Drip loss and cooking loss reflect the water-holding ability of muscles, and a higher water-holding ability represents better meat quality. Studies have shown that the rate of muscle drip loss is directly related to the cell membrane structure [38,39]. The addition of antioxidants to the diet prevents lipid peroxidation of the cell membrane and ensures its integrity, thereby preventing fluid outflow in the sarcoplasm and reducing the drip loss of muscles [40,41]. Therefore, a positive effect of antioxidation was associated with the improvement of meat quality.

Additionally, appropriate supplementation of Met in the diet results in lower lipid peroxidation, which is an important aspect of the spoilage process and volatilization of off odors [42,43]. Effectively reducing lipid peroxidation in the body may improve the odor and appearance of meat. There is a close relationship between the body’s antioxidant activity and muscle quality. Previous studies have demonstrated that the antioxidant activity of the body mainly depends on the formation of antioxidant enzymes and that antioxidant enzymes are closely related to the presence of selenium. Met also plays a very important role in improving the body’s antioxidant activity [10,11]. In the present study, supplementation with dl-Met or dl-Met-Met in the breeding pigeon diet increased the activities of T-SOD, GSH-Px and CAT and decreased the concentration of MDA in the muscles of squabs. This result was similar to that of Park and Zduńczyk et al., who found that supplementation with Met, regardless of its source, improved the T-SOD, GSH-Px and CAT activities of poultry muscle [13,44]. This is because Met residues can be combined with reactive oxygen species in the body to form two Met sulfoxide isomers, which regenerate Met under the action of Met sulfoxide reductase [16]. Our results demonstrated that the effect of dl-Met-Met was better than that of dl-Met. This may be mainly attributable to the fact that, compared with dl-Met, the previously described dl-Met-Met was easier to transport, was more absorbable across the brush border membrane and, on the other hand, was associated with greater antioxidant activity of the dipeptides [17,33,45]. To our surprise, the addition of dl-Met or dl-Met-Met did not affect the T-AOC. This might indicate that the supplemental dl-Met or dl-Met-Met improves the antioxidant activity of muscles mainly by increasing the activities of some antioxidant enzymes (CAT, T-SOD and GSH-Px) and then clearing reactive oxygen species or MDA in squabs [13]. However, the relationship between dietary Met and the T-AOC in the muscle still requires further investigation.

## 5. Conclusions

This study revealed that supplementation with 0.30% dl-Met and dl-Met-Met in breeding pigeons effectively improved the carcass characteristics, meat quality and antioxidant activity of squabs. Furthermore, a multiexponential regression equation indicated that dl-Met-Met can be used more efficiently than dl-Met as a source of Met for pigeons. Consequently, as antioxidants and nutrients, the increase in Met levels from different sources (especially dl-Met-Met) may provide a potential approach to improve the production and meat quality of squabs, and is significant for formulating the feeding standard of pigeons.

## Finding

The research was jointly supported by the National Natural Science Foundation of China (31972585; 31501969) and the Technical System of Poultry Industry of Guangdong Province, China (2019KJ128).

## Figures and Tables

**Table 1:** Compositions and nutrient levels of the experimental diets (air-dried basis, %).

Ingredient (%) ^1^	1	2	3	4	5	6	7	8	9
corn	51.00	51.00	51.00	51.00	51.00	51.00	51.00	51.00	51.00
soybean meal	17.50	17.50	17.50	17.50	17.50	17.50	17.50	17.50	17.50
wheat	24.00	24.00	24.00	24.00	24.00	24.00	24.00	24.00	24.00
sorghum	3.00	3.00	3.00	3.00	3.00	3.00	3.00	3.00	3.00
dicalcium phosphate	1.20	1.20	1.20	1.20	1.20	1.20	1.20	1.20	1.20
shell powder	1.00	1.00	1.00	1.00	1.00	1.00	1.00	1.00	1.00
salt	0.30	0.30	0.30	0.30	0.30	0.30	0.30	0.30	0.30
vitamin and mineral premix ^2^	1.00	1.00	1.00	1.00	1.00	1.00	1.00	1.00	1.00
lysine (98.5%)	0.40	0.40	0.40	0.40	0.40	0.40	0.40	0.40	0.40
dl-Met	0.00	0.15	0.30	0.45	0.60	0.00	0.00	0.00	0.00
dl-Met-Met	0.00	0.00	0.00	0.00	0.00	0.15	0.30	0.45	0.60
zeolite powder	0.60	0.45	0.30	0.15	0.00	0.45	0.30	0.15	0.00
total	100.00	100.00	100.00	100.00	100.00	100.00	100.00	100.00	100.00
formulated nutrient composition (%)									
metabolic energy (ME, MJ/kg)	12.13	12.13	12.13	12.13	12.13	12.13	12.13	12.13	12.13
crude protein	15.00	15.00	15.00	15.00	15.00	15.00	15.00	15.00	15.00
calcium	0.76	0.76	0.76	0.76	0.76	0.76	0.76	0.76	0.76
total phosphorus	0.56	0.56	0.56	0.56	0.56	0.56	0.56	0.56	0.56
nonphytate phosphorus	0.32	0.32	0.32	0.32	0.32	0.32	0.32	0.32	0.32
dl-Met	0.25	0.40	0.55	0.70	0.85	0.25	0.25	0.25	0.25
dl-Met-Met	-	-	-	-	-	0.15	0.30	0.45	0.60
lysine	0.96	0.96	0.96	0.96	0.96	0.96	0.96	0.96	0.96

**^1^** 1: basal diet; 2: 0.15% dl-Met; 3: 0.30% dl-Met; 4: 0.45% dl-Met; 5: 0.60% dl-Met; 6: 0.15% dl-Met-Met; 7: 0.30% dl-Met-Met; 8: 0.45% dl-Met-Met; 9: 0.60% dl-Met-Met. (BD: basal diet; dl-Met: dl-methionine; dl-Met-Met: dl-methionyl-dl-methionine); **^2^** Provided per kilogram of diet: vitamin A, 4000.00 IU; vitamin D_3_, 1725.00 IU; vitamin E, 24.00 mg; vitamin K_3_, 1.00 mg; vitamin B_1_, 3.00 mg; vitamin B_2_, 13.00 mg; vitamin B_6_, 2.00 mg; vitamin B_12_, 25.00 μg; niacin, 15.00 mg; folic acid, 0.55 mg; pantothenic acid, 7.50 mg; biotin, 0.12 mg; choline chloride, 200.00 mg; copper, 10.00 mg; iron, 35.00 mg; manganese, 55.00 mg; zinc 35.00 mg; iodine, 0.20 mg; selenium, 0.25 mg.

**Table 2:** Effects of breeding pigeon dietary supplementation with dl-Met or dl-Met-Met on the carcass characteristics of squabs (%)^1^.

Items^2^	Met Source	Met Dose	Carcass Yield	Semi-Eviscerated Yield	Eviscerated Yield	Breast Muscle Yield	Leg Muscle Yield
1	BD	0	84.38^e^	80.83^d^	64.41^d^	22.60^c^	6.83^c^
2	dl-Met	0.15%	84.86^de^	80.92^cd^	66.00^bc^	23.79^bc^	7.13^bc^
3	dl-Met	0.30%	86.39^ab^	82.27^a^	66.45^bc^	24.86^ab^	7.83^a^
4	dl-Met	0.45%	86.26^ab^	81.87^ab^	66.68^abc^	24.58^ab^	7.54^ab^
5	dl-Met	0.60%	85.07^cde^	81.23^bcd^	65.23^cd^	24.52^ab^	7.78^a^
6	dl-Met-Met	0.15%	85.99^bcd^	81.72^abc^	67.19^ab^	24.74^ab^	7.48^ab^
7	dl-Met-Met	0.30%	87.37^a^	82.27^a^	68.06^a^	26.07^a^	7.97^a^
8	dl-Met-Met	0.45%	86.40^ab^	82.16^a^	67.52^ab^	25.97^a^	7.90^a^
9	dl-Met-Met	0.60%	86.08^bc^	82.14^a^	67.22^ab^	25.76^a^	7.97^a^
SEM			0.175	0.118	0.226	0.241	0.078
*P*-values							
treatment effects			<0.001	0.002	<0.001	0.007	<0.001
met source			0.014	0.039	<0.001	0.010	0.084
main effect of the Met source							
dl-Met			85.64^b^	81.58^b^	66.09^b^	24.43^b^	7.57
dl-Met-Met			86.46^a^	82.07^a^	67.50^a^	25.64^a^	7.83
linear effect^3^							
dl-Met			0.072	0.131	0.180	0.015	0.002
dl-Met-Met			0.005	<0.001	0.001	0.002	<0.001
quadratic effect^3^							
dl-Met			0.008	0.023	0.004	0.076	0.173
dl-Met-Met			<0.001	0.006	<0.001	0.023	0.005

^1^ Values without the same small letters in the column indicate a significant difference (*p* < 0.05, n = 36); SEM = standard error of the mean; 2 1: BD; ^2^: 0.15% dl-Met; 3: 0.30% dl-Met; 4: 0.45% dl-Met; 5: 0.60% dl-Met; 6: 0.15% dl-Met-Met; 7: 0.30% dl-Met-Met; 8: 0.45% dl-Met-Met; 9: 0.60% dl-Met-Met. (BD: basal diet; dl-Met: dl-methionine; dl-Met-Met: dl-methionyl- dl-methionine); ^3^ Trend analysis for BD and BD with 0.15%, 0.30%, 0.45% and 0.60% dl-Met or dl-Met-Met.

**Table 3:** Effects of breeding pigeon dietary supplementation with dl-Met or dl-Met-Met on the meat quality of squabs (%)^1,2^.

Items^3^	Met Source	Met Dose	L*	a*	b*	Shear Force (N)	Drip Loss (%)	Cooking Loss (%)
1	BD	0	39.22^a^	19.40^c^	7.65	23.11	3.75^a^	26.86^a^
2	dl-Met	0.15%	38.67^ab^	21.31^b^	7.95	22.95	3.15^ab^	26.23^ab^
3	dl-Met	0.30%	36.33^c^	22.22^ab^	7.63	22.13	2.41^c^	23.32^e^
4	dl-Met	0.45%	36.46^c^	22.10^ab^	7.88	22.25	2.86^bc^	24.62^cd^
5	dl-Met	0.60%	37.28^bc^	22.03^ab^	7.97	22.49	3.17^ab^	25.64^bc^
6	dl-Met-Met	0.15%	37.97^abc^	21.69^ab^	7.82	21.73	2.85^bc^	25.98^ab^
7	dl-Met-Met	0.30%	36.89^c^	22.73^a^	7.66	21.61	2.37^c^	21.93^f^
8	dl-Met-Met	0.45%	37.03^bc^	21.95^ab^	7.95	22.37	2.49^c^	24.49^d^
9	dl-Met-Met	0.60%	37.65^abc^	21.32^b^	7.79	22.98	2.71^bc^	25.49^bcd^
SEM			0.214	0.214	0.103	0.434	0.082	0.167
*P*-values								
treatment effects			0.009	<0.001	0.993	0.996	<0.001	<0.001
Met source			0.636	0.990	0.801	0.768	0.023	0.327
main effect of the Met source								
dl-Met			37.18	21.91	7.86	22.45	2.90^a^	24.95
dl-Met-Met			37.39	21.92	7.80	22.17	2.60^b^	24.47
linear effect^4)^								
dl-Met			0.008	<0.001	0.552	0.663	0.069	0.023
dl-Met-Met			0.010	0.004	0.725	0.929	0.003	0.253
quadratic effect^4^								
dl-Met			0.049	0.012	0.896	0.746	0.002	<0.001
dl-Met-Met			0.008	<0.001	0.876	0.314	0.004	<0.001

^1^ Values without the same small letters in the column indicate a significant difference (*p* < 0.05, n = 36); SEM = standard error of the mean; ^2^ a* = redness, b* = yellowness, L* = lightness; ^3^ 1: BD; 2: 0.15% dl-Met; 3: 0.30% dl-Met; 4: 0.45% dl-Met; 5: 0.60% dl-Met; 6: 0.15% dl-Met-Met; 7: 0.30% dl-Met-Met; 8: 0.45% dl-Met-Met; 9: 0.60% dl-Met-Met. (BD: basal diet; dl-Met: dl-methionine; dl-Met-Met: dl-methionyl-dl-methionine); ^4^ Trend analysis for BD and BD with 0.15%, 0.30%, 0.45% and 0.60% dl-Met or dl-Met-Met.

**Table 4:** Effects of breeding pigeon dietary supplementation with dl-Met or dl-Met-Met on the antioxidant activity of the breast muscle of squabs^1,2^.

Items^3^	Met Source	Met Dose	CAT (U/mg-prot)	T-SOD (U/mg-prot)	GSH-Px (U/mg-prot)	T-AOC (U/mg-prot)	MDA (nmol/mg-prot)
1	BD	0	0.71^d^	18.94^e^	30.57^c^	0.35	0.86^a^
2	dl-Met	0.15%	0.77^c^	23.15^d^	35.88^bc^	0.41	0.70^b^
3	dl-Met	0.30%	0.81^abc^	28.43^ab^	38.34^ab^	0.46	0.59^b^
4	dl-Met	0.45%	0.81^abc^	26.68^abcd^	37.54^ab^	0.45	0.61^b^
5	dl-Met	0.60%	0.80^abc^	25.77^bcd^	38.01^ab^	0.44	0.56^b^
6	dl-Met-Met	0.15%	0.79^bc^	24.54^cd^	36.18^b^	0.41	0.64^b^
7	dl-Met-Met	0.30%	0.85^a^	25.77^a^	42.66^a^	0.44	0.59^b^
8	dl-Met-Met	0.45%	0.84^ab^	27.83^abc^	39.38^ab^	0.42	0.64^b^
9	dl-Met-Met	0.60%	0.84^ab^	28.20^ab^	40.51^ab^	0.44	0.65^b^
SEM			0.009	0.641	0.765	0.012	0.021
*P*-values							
treatment effects			<0.001	<0.001	0.008	0.604	0.013
Met source			0.032	0.159	0.117	0.636	0.653
main effect of the Met source							
dl-Met			0.80^b^	26.01	37.44	0.44	0.61
dl-Met-Met			0.83^a^	27.54	39.69	0.43	0.63
linear effect^4^							
dl-Met			0.003	<0.001	0.013	0.111	<0.001
dl-Met-Met			<0.001	<0.001	<0.001	0.068	0.027
quadratic effect^4^							
dl-Met			0.041	0.002	0.083	0.204	0.041
dl-Met-Met			0.003	0.002	0.009	0.276	0.017

^1^ Values without the same small letters in the column indicate a significant difference (*p* < 0.05, n = 8). The data are presented as the mean ± standard error; ^2^ CAT: catalase; T-SOD: total superoxide dismutase; GSH-Px: glutathione peroxidase; T-AOC: total antioxidant capacity; MDA: malonaldehyde; ^3^ 1: BD; 2: 0.15% dl-Met; 3: 0.30% dl-Met; 4: 0.45% dl-Met; 5: 0.60% dl-Met; 6: 0.15% dl-Met-Met; 7: 0.30% dl-Met-Met; 8: 0.45% dl-Met-Met; 9: 0.60% dl-Met-Met. (BD: basal diet; dl-Met: dl-methionine; dl-Met-Met: dl-methionyl- dl-methionine); ^4^ Trend analysis for BD and BD with 0.15%, 0.30%, 0.45% and 0.60% dl-Met or dl-Met-Met.

**Table 5:** Effects of breeding pigeon dietary supplementation with dl-Met or dl-Met-Met on the antioxidant activity of the thigh muscle of squabs^1,2^.

Items^3^	Met Source	Met Dose	CAT (U/mg-prot)	T-SOD (U/mg-prot)	GSH-Px (U/mg-prot)	T-AOC (U/mg-prot)	MDA (nmol/mg-prot)
1	BD	0	0.70	20.54^b^	31.84^b^	0.40	0.84^a^
2	dl-Met	0.15%	0.79	24.36^ab^	37.81^ab^	0.44	0.78^ab^
3	dl-Met	0.30%	0.79	27.28^a^	39.99^a^	0.44	0.69^bc^
4	dl-Met	0.45%	0.78	26.98^a^	39.25^a^	0.43	0.70^bc^
5	dl-Met	0.60%	0.81	28.29^a^	38.96^a^	0.46	0.66^c^
6	dl-Met-Met	0.15%	0.77	26.20^a^	39.01^a^	0.45	0.72^bc^
7	dl-Met-Met	0.30%	0.80	28.66^a^	43.11^a^	0.44	0.70^bc^
8	dl-Met-Met	0.45%	0.79	28.38^a^	41.17^a^	0.45	0.70^bc^
9	dl-Met-Met	0.60%	0.84	28.36^a^	42.43^a^	0.44	0.66^c^
SEM			0.010	0.643	0.808	0.008	0.013
*P*-values							
treatment effects			0.104	0.034	0.032	0.926	0.019
Met source			0.707	0.324	0.147	0.813	0.689
main effect of the Met source							
dl-Met			0.79	26.73	39.25	0.44	0.71
dl-Met-Met			0.80	27.90	41.43	0.45	0.70
linear effect^4^							
dl-Met			0.025	0.004	0.048	0.296	0.005
dl-Met-Met			0.015	0.003	<0.001	0.305	0.005
quadratic effect^4^							
dl-Met			0.163	0.216	0.061	0.668	0.202
dl-Met-Met			0.445	0.029	0.012	0.277	0.176

^1^ Values without the same small letters in the column indicate a significant difference (*p* < 0.05, n = 8). The data are presented as the mean ± standard error; ^2^ CAT: catalase; T-SOD: total superoxide dismutase; GSH-Px: glutathione peroxidase; T-AOC: total antioxidant capacity; MDA: malonaldehyde; ^3^ 1: BD; 2: 0.15% dl-Met; 3: 0.30% dl-Met; 4: 0.45% dl-Met; 5: 0.60% dl-Met; 6: 0.15% dl-Met-Met; 7: 0.30% dl-Met-Met; 8: 0.45% dl-Met-Met; 9: 0.60% dl-Met-Met. (BD: basal diet; dl-Met: dl-methionine; dl-Met-Met: dl-methionyl- dl-methionine); ^4^ Trend analysis for BD and BD with 0.15%, 0.30%, 0.45% and 0.60% dl-Met or dl-Met-Met.
